# Intra-Abdominal Hypertension and Its Prognostic Impact on Mortality in Cirrhotic Patients with Ascites: The Role of Paracentesis

**DOI:** 10.5152/tjg.2025.24375

**Published:** 2025-01-13

**Authors:** Ummu Mutlu, Sezen Genc Ulucecen, Raim Iliaz, Alp Atasoy, Bilger Cavus, Asli Ciftcibasi Ormeci, Filiz Akyuz, Kadir Demir, Sabahattin Kaymakoglu, Fatih Besisik

**Affiliations:** 1 Division of Endocrinology and Metabolism, Department of Internal Medicine, İstanbul University İstanbul Faculty of Medicine, İstanbul, Türkiye; 2 Division of Gastroenterohepatology, Department of Internal Medicine, İstanbul University İstanbul Faculty of Medicine, İstanbul, Türkiye

**Keywords:** Acute-on-chronic liver failure, ascites, cirrhosis, intra-abdominal hypertension, intra-abdominal pressure

## Abstract

**Background/Aims:**

Elevated intra-abdominal pressure (IAP) can lead to intra-abdominal hypertension (IAH) and, in severe cases, abdominal compartment syndrome (ACS) in patients with cirrhosis and ascites. Paracentesis reduces IAP and improves abdominal perfusion. Intra-abdominal hypertension can also trigger acute-on-chronic liver failure (ACLF) in decompensated cirrhosis. This study evaluates the association between IAH and short-term mortality in patients with cirrhosis and ascites.

**Materials and Methods:**

This prospective, single-center cohort study included 18 patients (7 females, 11 males; median age: 59) scheduled for therapeutic paracentesis. Intra-abdominal pressure was measured using the bladder technique. Patients were grouped based on initial Chronic Liver Failure Consortium Organ Failure (CLIF-C OF) scores as ACLF or non-ACLF and followed up for 3 months.

**Results:**

The median model for end-stage liver disease score was 17 (IQR 11-19). The primary etiologies of cirrhosis were viral hepatitis and alcoholic liver disease. Independent risk factors for IAH included advanced liver disease and large-volume ascites. Pre-paracentesis IAP was higher in ACLF patients (22 vs. 18 mm Hg). Post-paracentesis IAP was also higher in ACLF patients (14 vs. 8 mm Hg, *P* = .007). The 3-month mortality rate was 50%, with worse survival in ACLF patients (24 vs. 76.9 days, *P* = .002). Pre-paracentesis IAP was significantly higher in patients who died (22 vs. 18 mm Hg, *P* = .034), and survival was worse in those with IAP ≥18.5 mm Hg (*P* = .026).

**Conclusion:**

Intra-abdominal pressure is elevated in cirrhosis patients with grade 3 ascites. Despite similar paracentesis volumes, IAP remained higher in the ACLF group. Intra-abdominal pressure ≥18.5 mm Hg is associated with significantly reduced survival, indicating that IAH accelerates short-term mortality in these patients.

**Main Points**

- Intra-abdominal pressure (IAP) is the steady pressure of the abdominal cavity, and ascites increase IAP.
- In critically ill patients, IAP >12 mm Hg indicates intra-abdominal hypertension (IAH) and IAH is directly related to mortality.
- In our study, we aimed to investigate the effect of pre- and post-paracentesis IAP on prognosis in 18 cirrhotic patients with grade 3 ascites.
- Mortality significantly increased when IAP exceeded 18.5 mm Hg before paracentesis in the present study. Additionally, IAP levels were higher both pre- and post-paracentesis in patients with acute-on-chronic liver failure.
- Although the importance of IAP in intensive care patients is well-established, to our knowledge, this is the first study to examine its significance in cirrhotic patients with grade 3 ascites requiring intermittent paracentesis.

## Introduction

Intra-abdominal pressure (IAP) is a critical parameter in patients with liver cirrhosis and ascites. When IAP rises above 12 mm Hg, it leads to intra-abdominal hypertension (IAH), and in more severe cases, it can progress to abdominal compartment syndrome (ACS), which is associated with potential organ failure. Liver cirrhosis and ascites are significant risk factors for both IAH and ACS. Paracentesis plays a crucial role in managing IAH by reducing abdominal wall tension, improving intra-abdominal hemodynamics, minimizing IAP, and optimizing abdominal perfusion pressure of the abdominal organs. Therefore, therapeutic paracentesis involves the removal of at least 5 liters of fluid, aiming to reduce IAP and alleviate symptoms such as shortness of breath, abdominal discomfort, and early satiety.^1^^,^^2^

Since IAH develops slowly in patients with chronic liver disease and ascites, the abdominal wall can adapt to the increase in IAP. The increased flexibility of the abdominal wall over time is called the stress-relaxation phenomenon; thus, the effect of the increase in IAP on organs is better compensated than in other conditions. In patients with chronic ascites, as in liver cirrhosis, the IAP is observed only when IAP is above 25 mm Hg and the findings can be eliminated by paracentesis.^3^^-^^5^

Intra-abdominal pressure is now measured and monitored in all intensive care patients with risk factors. Cirrhosis and grade 3 ascites are also risk factors for IAH.^2^ Intra-abdominal hypertension is a condition that disrupts gastrointestinal barrier functions and causes a predisposition for organ failure in acute events. In patients with grade 3 ascites, the IAH may initiate acute-on-chronic liver failure (ACLF) and adversely affect prognosis. Hepatic artery and portal system blood flow decreases due to increased IAP. In some patients, acute liver failure may also develop.^6^ Mesenteric hypoperfusion causes intestinal edema, ischemia, and bacterial translocation. Sepsis could be seen in these patients secondary to bacterial translocation.^7^^,^^8^

Studies evaluating the impact of IAP and IAH on mortality and morbidity in decompensated cirrhosis patients with grade 3 ascites requiring paracentesis are limited.^1^^,^^9^^,^^10^ However, some studies have shown that IAH is associated with organ dysfunction and failure.^11^ The aim of the present study is to evaluate the relationship between grade 3 ascites and IAH, whether IAH is a risk factor for ACLF, and its association with short-term mortality prospectively.

## Materials and Methods

### Ethics

This study was performed following the Helsinki recommendations. Written informed consent was obtained from patients. This study was performed with the Institutional Review Board of İstanbul University İstanbul Faculty of Medicine protocol approval dated February 21, 2017, and number 194.

### Subjects

This is a single-center prospective cohort study conducted in outpatient and inpatient clinics of İstanbul University, İstanbul Faculty of Medicine, Department of Internal Medicine. Sixty patients aged 18 years or older with a diagnosis of cirrhosis, with grade 3 ascites at least at the umbilicus level, planned for paracentesis, and who gave consent to participate in the study were evaluated from August 2016 to January 2017. Clinical and laboratory features, pathological examination, and imaging methods used to diagnose cirrhosis.

**Figure 1.**
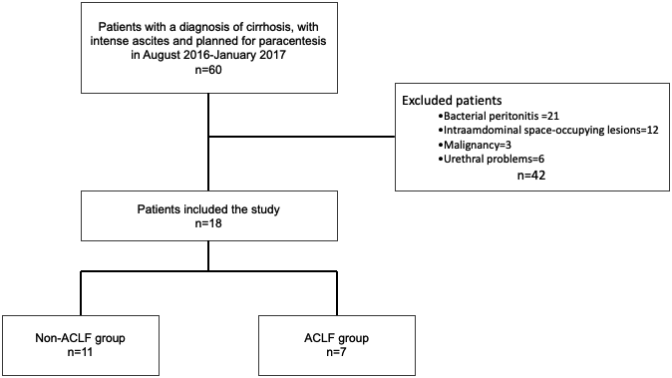
Flowchart of the study population. ACLF, acute-on-chronic liver failure.

Patients with bacterial peritonitis, intra-abdominal space-occupying lesions, decompensated with acute ascites, muscle disorders that may affect respiratory muscles, anatomical problems or urinary infections that prevent Foley catheter insertion, and active alcohol users were excluded from the study. Eighteen patients were included (Figure 1).

The attending physician determined the indications for paracentesis. Intra-abdominal pressure was measured using the bladder technique in patients who consented to participate in the study. In this method, a urinary catheter is inserted into the bladder and clamped before measurement. An 18-Gauge needle is inserted into the sampling port and connected to the transducer. A sterile saline infusion bag is attached to one end of the 2-way transducer and 25-50 cc of saline is infused into the bladder through a 20 mL syringe. The transducer cable is connected to the monitor, and the measurement is made by resetting at the level of the iliac crest in the mid-axillary line. Intra-abdominal pressure is measured with the patient in a supine position at the end of expiration.^1^^,^^2^

After the measurement, paracentesis was performed. The amount of paracentesis performed was recorded in liters for each patient, with large volume paracentesis being performed when necessary. Grade 3 ascites was defined according to the classification proposed by the International Ascites Club: grade 1 – mild ascites detectable only by ultrasound, grade 2 – moderate ascites with moderate symmetrical abdominal distension, and grade 3 – gross ascites with marked abdominal distension.^12^ Immediately after paracentesis, the patient’s IAP was measured again. The patients were followed for 3 months and evaluated in terms of mortality and complications of cirrhosis. In the beginning, the organ failure of the patients was calculated according to the Chronic Liver Failure Consortium Organ Failure (CLIF-C OF) scoring system, and they were also evaluated in 2 sub-groups; patients with ACLF and those without ACLF. The effects of high IAP on survival, complications, Model for End-stage Liver Disease (MELD), Model for End-stage Liver Disease-Sodium (MELD-Na), Child–Pugh scores, Chronic Liver Failure Consortium Acute decompensation/ACLF (CLIF-C AD/ACLF) were investigated.^13^^,^^14^ Chronic Liver Failure Consortium Acute Decompensation was used for the non-ACLF group, and CLIF-C ACLF was used for the ACLF group. In addition, subgroup analysis was performed as survivors and non-survivors to determine the risk factors predicting mortality.

### Statistical Analysis

Data were analyzed using IBM SPSS Statistics, version 21 (IBM SPSS Corp.; Armonk, NY, USA). The Kolmogorov–Smirnov test was used to assess normality, in addition to histograms and boxplots. Descriptive analyses were displayed using frequency tables for categorical variables, and non-normal distributions were presented as medians with interquartile ranges. The Mann–Whitney *U* test was performed to compare groups. Cross-group comparison for categorical variables was obtained using chi-square/Fisher tests. In correlation analysis, the Spearman test was used. A “*P*” value of <.05 was considered statistically significant. An receiver operating characteristics curve was performed to establish the cut-off value of pre-paracentesis IAP.

## Results

The demographic and descriptive data of the patients are summarized in Table 1. Among 18 patients, 38.9% (n = 7) were female, and the median age was 59 (IQR; 52-69). The distribution of patients according to cirrhosis etiology is also shown in Table 1. There was no active alcohol use in patients with alcoholic fatty liver disease. The median Child–Pugh and MELD scores were 15 (IQR 7-15) and 17 (IQR 11-19), respectively. Since IAP before paracentesis was above 12 mm Hg in all patients, all of them had IAH.

Seven of the 18 patients had acute chronic liver failure based on the CANONIC (Chronic liver failure acute-on-chronic liver failure) study results.^12^^,^^13^ Among these patients, 3 had hepatorenal syndrome, and 4 had hepatic encephalopathy. A subgroup analysis was performed between the ACLF and non-ACLF groups. No difference was found regarding pre-paracentesis IAP and the amount of paracentesis, respectively (*P* = .165; *P* = .280). However, post-paracentesis IAP was higher in the ACLF group than in the non-ACLF group (*P* = .007) (Table 1).

## Survival

Patients were evaluated for survival. Half of the patients died during the follow-up period. Age and gender were not associated with mortality (Table 2). Patients who were included in the cadaveric list due to lack of a suitable living donor were not able to undergo transplantation during the follow-up period. Ten patients with a MELD score >14 did not have a living donor. Three patients were not candidates for transplantation due to lack of social support, 3 due to advanced age and cardiopulmonary issues, and 2 due to portal thrombosis. The other 2 patients were followed on the cadaveric transplant list.

**Figure 2.**
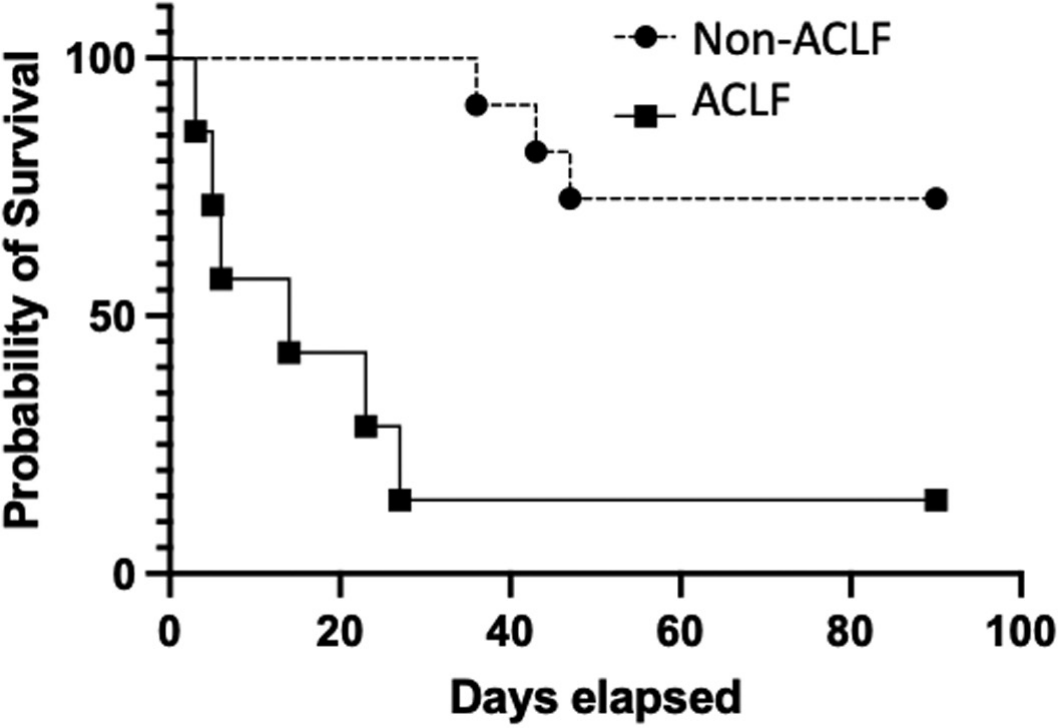
Survival of the ACLF and non-ACLF groups. ACLF, acute-on-chronic liver failure.

The mortality rate was higher in the ACLF group (respectively, 85.7% vs. 27.3%, *P* = 0.05) (Figure 2). The causes of death were pneumonia and sepsis in 2 patients, variceal bleeding in 1 patient, hepatic encephalopathy in 2 patients, spontaneous bacterial peritonitis (SBP) in 1 patient, and multiorgan failure due to hepatorenal syndrome in 3 patients.

No statistically significant difference was observed between the non-survivor and survivor patients regarding baseline Child–Pugh, MELD, MELD-Na, CLIF-C OF scores, or CLIF-C AD/ACLF parameters.

When the effect of IAP on mortality was examined, pre-paracentesis IAP was higher in patients who died than in survivors (22 vs. 18 mm Hg, *P* = .034). The median paracentesis volume was higher in survivors (Table 2).

There was a negative correlation between the CLIF-C OF, MELD, and MELD-Na scores and survival, while a positive correlation was detected with the amount of paracentesis (respectively: *r* = −0,533, *P* = .023; *r* = −0.534, *P* = .022; *r* = −0.456, *P* = .036; *r* = −0.528, *P* = .024; *r* = 0.608, *P* = .007). No correlation was detected between CLIF-C AD/ACLF scores and survival (*r* = −0.377, *P* = .123). There was no significant correlation between pre-paracentesis IAP, post-paracentesis IAP, and survival (*r* = −0.46, *P* = .054; *r* = −0.41, *P* = .089 respectively).

**Figure 3.**
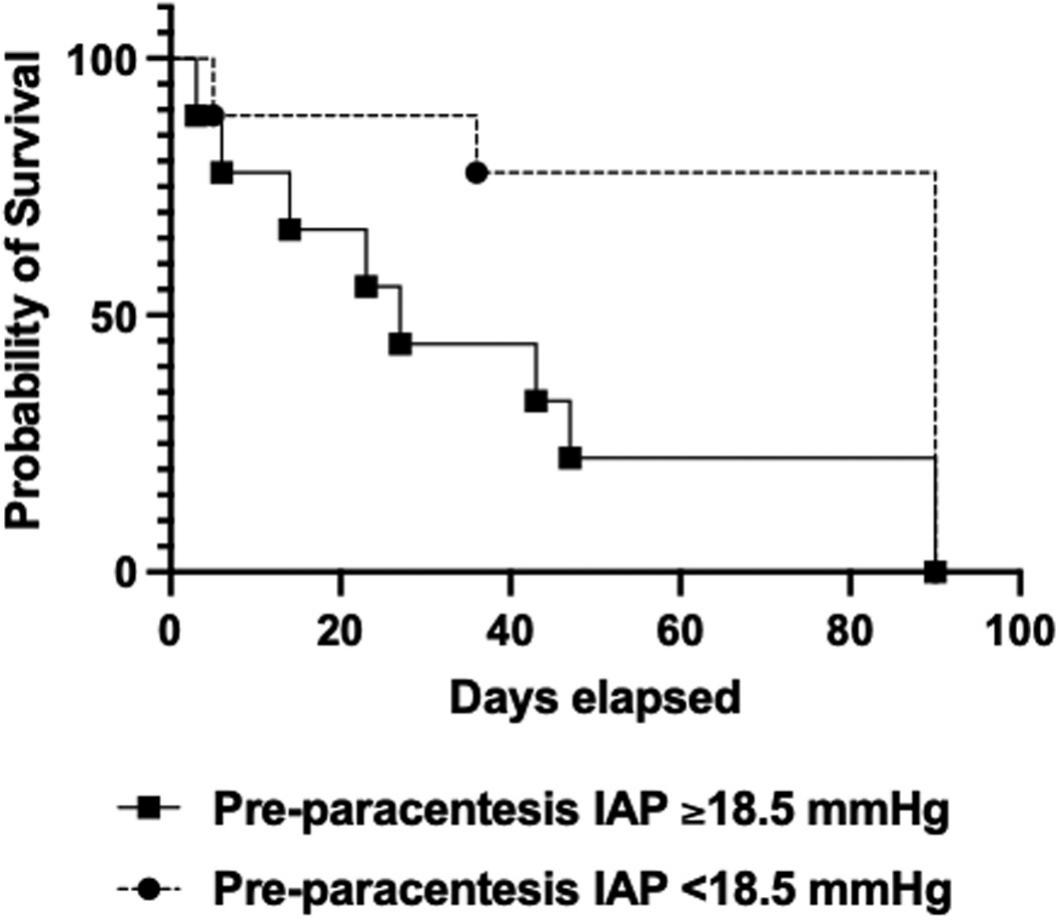
Survival of the patients according to pre-paracentesis IAP.

The cut-off value of the pre-paracentesis IAP was found to be 18.5 mm Hg in the ROC analysis with 77.8% sensitivity and 88% specificity, and the AUC (area under curve) value was 0.790 (95% CI, 0.572-1.000; *P* < .038). The survival was worse when pre-paracentesis IAP was ≥18.5 mm Hg (38.1 ± 10.4 days vs. 74.6 ± 9.9 days, *P* = .026) (Figure 3).

## Discussion

It is well-established that there are many factors affecting the prognosis of cirrhosis, such as etiology, disease severity, presence of complications, and comorbid diseases. Moreover, when decompensation develops, mortality increases dramatically.^6^^-^^8^ D’Amico et al^15^ found in their review of 118 studies that patients with compensated cirrhosis had a survival of over 12 years, while decompensated patients had approximately 2 years. Annual mortality was approximately 1% in compensated patients and 3.4% in compensated patients with esophageal varices. According to this review, 6.6% of patients per year decompensate with the development of ascites, 7.6% experience variceal bleeding, and the annual mortality is 20% in patients with ascites and 57% in patients with variceal bleeding (most deaths occur within the first 6 weeks after bleeding). As can be seen, the development of ascites significantly increases mortality.

In patients with cirrhosis, increased IAP leads to IAH. This is a predisposing condition for organ failure and increases mortality. The World Abdominal Compartment Syndrome Association (WSACS) defines IAH as an abdominal pressure above 12 mm Hg in repeated measurements. Abdominal Compartment Syndrome (ACS) is defined as IAP >20 mm Hg and signs of organ failure together.^2^ However, since the development of ascites in cirrhosis patients may be long-term, IAP may be higher than in other patients and ACS may not develop.^5^

Direct and indirect methods can be applied for the IAP measurement. During laparoscopy or laparotomy, a catheter is placed in the abdomen and IAP can be directly measured. It is not preferred because of the risk of contamination of the peritoneal cavity.^16^ Therefore, the IAP is measured indirectly.^17^^,^^18^ Clinical and experimental studies showed the correlation between the pressure in the stomach, bladder, rectum, and vena cava inferior and IAP.^19^^,20^ The gold standard method is the bladder technique because it is the least invasive and easy to apply. The only disadvantage is that it can cause urinary system infections.^21^ We also used the bladder technique in our study. None of the patients had a urinary infection or any other procedure-related complication.

Oliguria that can develop with increasing IAP was first described by Wendt^22^ in 1876, but there has not been much study on this subject afterward. In a study by Luca et al^5^, in 14 patients with portal hypertension, increasing IAP by 10 mm Hg decreased hepatic blood flow by 20%.^5^ Rasmussen et al^23^, showed that, when IAP was 25 mm Hg, portal vein blood flow decreased by 66%. Thus, increased IAP can contribute to hemodynamic instability and may play a role in the progression of ACLF. In another study, increased IAP may trigger variceal bleeding by causing an increase in varicose pressure.^24^ Similar to these studies, the majority of patients who died during the follow-up period in our study died due to complications of cirrhosis. In these patients, IAP was higher than 18.5 mm Hg.

Increased IAP can compromise gastrointestinal mucosal integrity, which plays a critical role in the development of ACLF through bacterial translocation and subsequent infections. Elevated IAP reduces mesenteric blood flow, leading to intestinal ischemia, which weakens the epithelial barrier. This disruption allows bacteria and endotoxins to enter systemic circulation, promoting septic events and triggering systemic inflammatory responses, both of which can exacerbate the progression of ACLF. Studies have highlighted the link between bacterial translocation and IAP, suggesting that these mechanisms contribute to poor outcomes in cirrhotic patients.^25^ Additionally, IAP can impair lymphatic drainage, reducing the body’s immune defenses and increasing the likelihood of infections such as SBP, a common and severe complication in cirrhotic patients.^26^ In our study, although we did not directly evaluate the incidence of infections, patients with IAP ≥18.5 mm Hg exhibited significantly higher short-term mortality. Moreover, 2 patients died due to pneumonia and sepsis, and 1 patient died from SBP in the present study. This finding aligns with the proposed mechanisms of bacterial translocation and infection contributing to the worsening of ACLF.

In a study conducted by Al-Dorzi et al^9^ in 61 cirrhosis patients hospitalized in the intensive care unit with septic shock, it was shown that IAH developed in 82% of patients on the first day and 97% of them in the following 7 days, and mortality was higher in patients with IAH. However, a correlation was not found between the development time of IAH and mortality. In our study, all patients had chronic grade 3 ascites and regular paracentesis (weekly or every 2 weeks) was performed. Despite this, all of them had IAH. No correlation was found between the frequency of paracentesis and IAH. In the study by Reintam Blaser et al^11^, similar to our study, in 240 cirrhosis patients followed in the intensive care unit, it was shown that the risk of mortality increases as the degree of IAH increases.

In the literature, studies investigating the effect of IAP on prognosis and mortality in cirrhotic patients with grade 3 ascites and its importance in patients with acute failure on chronic liver disease are limited. In a study conducted by Umgelter et al^10^ in 23 cirrhotic patients with hepatorenal syndrome hospitalized in the intensive care unit, it has been shown that IAP decreased from 22 mm Hg to 9 mm Hg on average and creatinine clearance increased from 23 mL/min to 33 mL/min with therapeutic paracentesis performed after infusion of 20% 200 mL human albumin. In our study, the mean IAP before paracentesis was 18.5 mm Hg (IQR; 18-22) and the mean IAP after paracentesis was 10 mm Hg (IQR; 8-12). However, unlike Umgelter et al’s study, although the amount of paracentesis performed was similar, when patients with ACLF were examined, pre-paracentesis IAP was 22 mm Hg, while post-paracentesis IAP was 14 mm Hg. Post-paracentesis IAP was found to be higher in the patient group with ACLF. This suggested that the persistence of high IAP after paracentesis in patients with ACLF is an ascites-independent parameter in IAH. Like the data in the literature, mortality was higher in patients with ACLF and high IAP.^1^ In a multicentric, retrospective study by Pereira et al,^1^ alcohol was shown to be an independent risk factor for IAH in 95 patients. We could not identify an etiological risk factor in our study, probably due to the small sample size. Since the patients with IAP measurement were awake, it should be considered that respiration and muscle tone may have slightly increased IAP. By following the other recommendations of the WSACS, changes that may occur in the IAP have been tried to be minimized.

Our study found that short-term mortality increased in decompensated cirrhotic patients with grade 3 ascites and elevated IAP. Additionally, survival was worse in patients with pre-paracentesis IAP of 18.5 mm Hg or higher, according to the survival ROC analysis. If confirmed by further studies, these findings could help establish the IAP threshold that indicates the need for paracentesis in cirrhotic patients. With more research, IAP may become a criterion for decompensation and a key target in patient management.

One of the major limitations of this study is the small sample size. While the study initially began with 60 patients, it was reduced to 18 patients due to strict exclusion criteria, which may have limited the statistical power of the findings. Among the excluded patients were those with active infections, such as SBP, as we aimed to create a more homogeneous sample to specifically evaluate the impact of IAH without the confounding effects of infection-related complications. Additionally, while infection is a critical factor in the progression of ACLF, excluding patients with active infections prevented us from evaluating its role in patient outcomes in this cohort. However, due to the limited number of studies on IAP measurement and IAH in patients with cirrhosis with grade 3 ascites, the normal distribution of the data, and our results being consistent with the published data.

In conclusion, IAH increases short-term mortality in patients with cirrhosis. Although similar amounts of paracentesis are performed in patients with ACLF, short-term mortality is higher, indicating that IAH contributes to mortality as an ascites-independent parameter. Mortality is significantly higher when IAP is ≥18.5 mm Hg. Conducting controlled, randomized studies with standardized paracentesis amounts in larger patient groups may more accurately reveal the impact of IAP on prognosis in this patient group.

## Figures and Tables

**Table 1.** Comparison of Demographic and Clinical Characteristics and IAP Measurements of Patient Groups with and Without ACLF

	**All Patients (n = 18)**	**ACLF (n = 7) **	**Non-ACLF (n = 11)**	P*
Demographic characteristics				
Age (median; IQR)	59 (52-69)	57 (49-69)	60 (52-69)	.495
Sex (n, female; %)	7 (38.9)	2 (28.6)	5 (45.4)	.637
Etiology of cirrhosis (n)				
Alcoholic fatty liver disease	5	4	1	
NAFLD	4	1	3	
Cryptogenic liver disease	3	0	3	
Hepatitis B, C	4	1	3	
Autoimmune hepatitis	1	1	0	
Budd Chiari	1	1	0	
Biochemical data (median; IQR)				
Creatinine (mg/dL)	1 (0.8-2.2)	1.6 (0.8-2.4)	0.8 (0.7-1.5)	.184
AST (U/L)	41 (32-54)	54 (24-129)	40 (33-48)	.204
ALT (U/L)	25 (22-33)	25 (18.5-63)	24 (22.3-31)	1.000
Albumin (g/dL)	2.89 (2.7-3.1)	2.9 (2.7-3.1)	2.86 (2.3-3.1)	.962
Bilirubin (mg/dL)	1.6 (0.9-2.5)	3.6 (1.2-5.8)	1.3 (0.9-2.2)	.283
INR	1.3 (1.2-1.5)	1.5(1.1-1.8)	1.3 (1.1-1.6)	.186
Disease severity (median; IQR)				
Child–Pugh score	15 (7-15)	11 (9-13)	9 (9-10)	.113
MELD score	17 (11-19)	19 (18-20)	11 (9-17)	**.009**
MELD-Na score	19 (16-24)	24 (20-28)	16 (14-19)	**.012**
CLIF-C OF score	7 (7-8)	8 (8-14)	7 (6-7)	**.001**
The frequency of paracentesis (days) (median; IQR)	15 (7-19)	7 (3-15)	15 (15-21)	**.025**
Pre-paracentesis IAP (mm Hg) (median; IQR)	18.5 (18-22)	22 (18-22)	18 (16-21)	.165
Post-paracentesis IAP (mm Hg) (median; IQR)	10 (8-12)	14 (11-14)	8 (7-11)	**.007**
Cirrhosis complications on follow-up, n (%)	12 (66)	6 (85.7)	6 (54.5)	.316
Mortality, n (%)	9 (50)	6 (85.7)	3 (27.3)	**.05**
90-day survival (mean ± SD)	56.3 ± 36.5	24 ± 30.5	76.9 ± 22	**<.001**

ACLF, acute-on-chronic liver failure; ALT, alanine transaminase; AST, aspartate transaminase; CLIF-C AD/ACLF, Chronic Liver Failure Consortium Acute decompensation/acute-on-chronic liver failure; CLIF-C OF, Chronic Liver Failure Consortium Organ Failure; IAP, intra-abdominal pressure; IQR, interquartile range; MELD, model for end-stage liver disease; MELD-Na, model for end-stage liver disease-sodium; NAFLD, non-alcoholic fatty liver disease.*Statistically significant P values (P<.05) are indicated in bold.

Statistically significant *P* values (*P <* .05) are indicated in bold.

**Table 2.** Demographic and Clinical Characteristics of Patients Regarding 90-day Mortality

	**Non-Survivors (n = 9)**	**Survivors (n = 9)**	P*
Demographics			
Age (year) median, IQR	61.5 (51.5-70.5)	59 (52-69)	.657
Sex (no. female, %)	2 (26.8)	5 (71.4)	.335
Biochemical data			
Creatinine (mg/dL)	1.3 (0.8-1.9)	0.8 (0.8-2.4)	.789
AST (U/L)	58 (38.5-96)	39 (32-41)	.750
ALT (U/L)	25.2(18.5-67)	25 (22.6-29.2)	.810
Albumine (g/dL)	2.88 (2.66-3)	2.9 (2.4-3.1)	.691
Bilirubin (mg/dL)	2.8(1.1-5.8)	1 (0.9-1.9)	.171
INR	1.38 (1.2-1.8)	1.3 (1.2-1.3)	.426
Baseline disease severity			
Child–Pugh score	11 (9-12)	9 (9-10)	.084
MELD score	18.5 (16.5-19.5)	11 (9-19)	.182
MELD-Na score	20.5(18-24)	16 (14-19)	.098
CLIF-C OF score	8 (7-11.5)	7 (7-7)	.218
The frequency of paracentesis (days)(median; IQR)	15 (7-15)	15 (7-21)	.363
Pre-paracentesis IAP (mm Hg)	22 (19-22)	18 (16-18)	**.034**
Patients with pre-paracentesis IAP ≥ 18.5 mm Hg %, (n)	77.8 (7)	22.2 (2)	.057
Post-paracentesis IAP (mm Hg)	11 (9-14)	8 (7-12)	.087

ALT, alanine transaminase; AST, aspartate transaminase; CLIF-C AD/ACLF, Chronic Liver Failure Consortium Acute decompensation/acute-on-chronic liver failure; CLIF-C OF, Chronic Liver Failure Consortium Organ Failure; IQR, interquartile range; MELD, model for end-stage liver disease; MELD-Na, model for end-stage liver disease-sodium.*Statistically significant P values (P<.05) are indicated in bold.

## Data Availability

The data that support the findings of this study are available on request from the corresponding author.
